# Operando double-edge high-resolution X-ray absorption spectroscopy study of BiVO_4_ photoanodes

**DOI:** 10.1107/S1600577524002741

**Published:** 2024-04-15

**Authors:** Alberto Piccioni, Jagadesh Kopula Kesavan, Lucia Amidani, Raffaello Mazzaro, Serena Berardi, Stefano Caramori, Luca Pasquini, Federico Boscherini

**Affiliations:** a Department of Physics and Astronomy, Alma Mater Studiorum – Università di Bologna, Viale C. Berti Pichat 6/2, 40127 Bologna, Italy; b The Rossendorf Beamline at the European Synchrotron Radiation Facility, Grenoble, France; c Institute of Resourse Ecology, Helmholtz-Zentrum Dresden-Rossendorf, Dresden, Germany; d Dipartimento di Chimica e Scienze Parafarmaceutiche, Università di Ferrara, Italy; ESRF – The European Synchrotron, France

**Keywords:** X-ray absorption spectroscopy, photoelectrochemistry, photoanodes, resonant inelastic X-ray scattering

## Abstract

High energy resolution fluorescence detected X-ray absorption spectroscopy is a powerful method for probing the electronic structure of functional materials. The X-ray penetration depth and photon-in/photon-out nature of the method allow operando experiments to be performed, in particular in electrochemical cells. Here, operando high-resolution X-ray absorption measurements of a BiVO_4_ photoanode are reported, simultaneously probing the local electronic states of both cations. Small but significant variations of the spectral lineshapes induced by the applied potential were observed and an explanation in terms of the occupation of electronic states at or near the band edges is proposed.

## Abstract 2

Operando high-resolution X-ray absorption spectroscopy of BiVO_4_ photoanodes was performed in an electrochemical cell at both cation absorption edges. Small but significant variations of the spectra induced by electrochemical polarization were detected which were interpreted in terms of changes in the occupation of electronic states.

## 1. Introduction

### 1.1. X-ray absorption and related techniques in the study of materials for photoelectrochemistry

X-ray absorption fine structure (XAFS) and related techniques (Van Bokhoven & Lamberti, 2016 ▸; International Tables for Crystallography, 2024 ▸) are powerful tools in the study of advanced functional materials. Analysis of the near-edge and extended part of the spectrum (XANES and EXAFS, respectively) can provide element-specific information on the local atomic and electronic structure. Analysis of the spectrum of resonantly inelastically scattered photons has given rise to the high energy resolution fluorescence detected–X-ray absorption near-edge structure (HERFD–XANES) method (Glatzel & Bergmann, 2005 ▸) which often has greater sensitivity to small changes in the local structure than XAFS. Of particular interest is that these methods can be applied in operando conditions in order to detect changes in the local structure induced by variations of the operating parameters of a device. For example, HERFD–XANES has been used to study illumination-induced charge transfer processes in TiO_2_ sensitized by Au nanoparticles (Amidani *et al.*, 2015 ▸) and in V-doped TiO_2_ (Rossi *et al.*, 2017 ▸).

There is intense current research in materials at the base of photoelectrochemical processes and devices for applications such as water splitting and contaminant degradation. XAFS and related techniques can be used to obtain atomistic insight on the fundamental physical and chemical processes in this field, very useful in order to go beyond a trial-and-error approach in materials optimization, as reported by some recent reviews (Fabbri *et al.*, 2017 ▸; Fracchia *et al.*, 2018 ▸; Minguzzi & Ghigna, 2017 ▸). For example, Braun *et al.* (2012 ▸) have studied hematite photoanodes with O *K*-edge XANES, Ir and Ir oxide electrodes and heterojunctions have been studied by several groups (Minguzzi *et al.*, 2013 ▸, 2017 ▸; Abbott *et al.*, 2016 ▸; Pfeifer *et al.*, 2017 ▸) as also mixed Ni and Fe oxyhydrides (Friebel *et al.*, 2015 ▸; Görlin *et al.*, 2016 ▸).

BiVO_4_ is an n-type semiconductor with an intermediate energy gap possessing several interesting properties for photoelectrochemical applications. BiVO_4_ can be obtained in the form of thin films via a variety of potentially scalable methods involving relatively cheap and abundant chemical precursors. It absorbs light up to 500 nm with direct optical transitions, displays a band alignment suitable for driving many relevant redox reactions and it is moderately stable in aqueous electrolytes under anodic polarization. In this paper, we report and discuss operando HERFD–XANES measurement of porous BiVO_4_ photoanodes at both the V *K*- and Bi *L*_III_-edges, highlighting how the method allows the detection of small but significant changes in the occupation of electronic states induced by electrochemical polarization with elemental sensitivity.

### 1.2. Atomic and electronic structure of BiVO_4_ and related materials

Sayama *et al.* (2006 ▸) deposited porous BiVO_4_ thin films by metal-organic decomposition and studied their properties by a combination of experimental and computational methods. Their films consisted of nanoparticles of diameter 90–150 nm exhibiting the monoclinic scheelite-type crystal structure. In this structure vanadium and bis­muth are found in a distorted tetrahedron and dodecahedron, respectively; the formal oxidation states are Bi^3+^ (6*s* ^2^), V^5+^ (3*d* ^0^) and O^2−^ (2*p* ^6^). The band structure was studied by photoelectron spectroscopy combined with density functional theory (DFT) calculations. A very detailed study of the electronic structure of monoclinic BiVO_4_ was reported later (Cooper *et al.*, 2014 ▸), with a combination of DFT simulations, photoemission, XAS, XES and RIXS. These authors concluded that the top of the valence band originates from unhybridized O 2*p*_π_ and Bi 6*s* orbitals and the bottom of the conduction band from V 3*d* orbitals hybridized with antibonding O 2*sp* ^2*^ ones, which are split into three levels by lattice distortions. The states at the bottom of the conduction band are strongly localized on V. The band structure of BiVO_4_ has been compared with that of other photocatalytic materials by Sivula & van de Krol (2016 ▸).

Previous XAFS studies of compounds related to BiVO_4_ have been reported. Concerning the vanadium *K*-edge, we note an early detailed study (Bianconi, 1982 ▸) of the pre-edge features in the V *K*-edge spectra of VO_2_ across the metal-to-insulator transition and systematic studies of the variation of the pre-edge and XANES features in V compounds, minerals, and basaltic and silicate glasses (Wong *et al.*, 1984 ▸; Giuli *et al.*, 2004 ▸; Sutton *et al.*, 2005 ▸) and an extensive study also using *ab initio* simulations (Bordage, 2010 ▸). More recently, a comparison of the spectra of several model compounds with full multiple scattering simulations has been published (Benzi *et al.*, 2016 ▸). These papers demonstrate the dependence of spectral features on local coordination and oxidation state. In particular, with increasing oxidation state the energy of the pre-edge peak and its intensity clearly increase.

As for XAFS spectra at the Bi *L*_III_-edge, an early study has been published by Jiang & Spence (2006 ▸). A thorough review of Bi *L*_I_ and *L*_III_ HERFD–XANES spectra of several Bi compounds and a comparison with *ab initio* simulations of the spectra and density of states using the *FDMNES* code (Joly, 2001 ▸) has been reported by Mistonov *et al.* (2018 ▸). Metallic Bi, α-Bi_2_O_3_, BiPO_4_, Bi_4_(GeO_4_)_3_ and NaBiO_3_ were studied. They observed that both the oxidation state and the length of the Bi–ligand bonds determine the exact position of the absorption edge. Additionally, thanks to the high energy resolution, spectral features corresponding to strong Bi *p*–*d* orbital mixing were resolved. At the *L*_III_-edge, the pre-edge feature is due to quadrupole transitions to Bi *p* states, which is a general finding for these compounds, and the main edge features correspond to Bi *d* states; the usual *t*_2g_–*e*_g_ crystal field splitting is also observable.

## 2. Experimental

WO_3_/BiVO_4_ photoanodes were deposited as described previously (Cristino *et al.*, 2016 ▸, 2019 ▸). Nanoporous BiVO_4_ layers were deposited by a potentiostatic procedure on WO_3_ films deposited on fluorine-doped tin oxide (FTO) substrates. Briefly, 10 m*M* VOSO_4_ in Millipore water was acidified by addition of HNO_3_ up to pH 0.5 followed by the addition of 10 m*M* Bi(NO_3_)_3_. Further HNO_3_ was added until Bi(NO_3_)_3_ was completely dissolved. Subsequently, the pH was quickly increased to 4.5 by using 2 *M* CH_3_COONa. This solution was immediately used for two electrode potentiostatic electrodepositions by applying 210 mV between FTO/WO_3_ and a platinum foil for 600 s at room temperature. The resulting substrates were thoroughly rinsed with Millipore water and annealed at 500°C for 2 h in order to form nanocrystalline BiVO_4_ in a monoclinic form. The crystallographic structure and morphology of the photoanodes as well as their photoelectrochemical response were reported in a previous publication (Cristino *et al.*, 2019 ▸).

**Figure 1:**
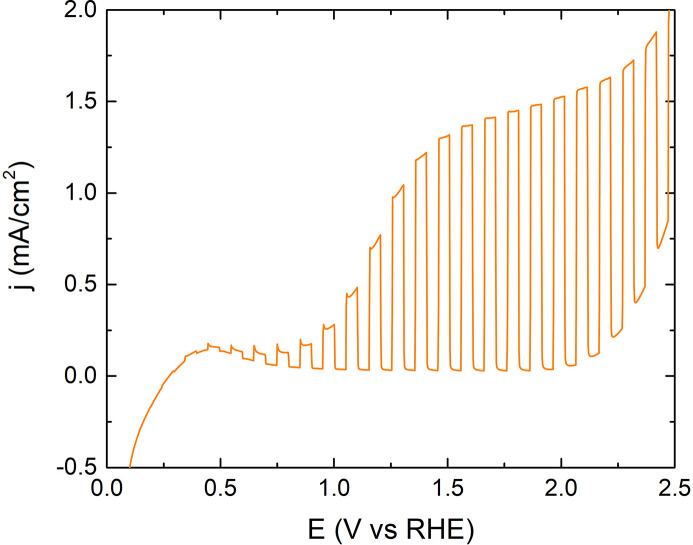
LSV recorded in aqueous 0.5 *M* Na_2_SO_4_ at neutral pH under chopped AM 1.5 G illumination.

In Fig. 1 ▸ we report linear sweep voltammetry (LSV) measured in aqueous 0.5 *M* Na_2_SO_4_ at neutral pH under chopped AM 1.5 G illumination. The LSV displays the typical n-type oxide behaviour, in which the photocurrent increases with the anodic bias up to a plateau current of 1.4 mA cm^−2^ [above 1.6 V versus reversible hydrogen electrode (RHE)]. In the absence of illumination (dark), the current is still negligible at 1.85 V versus RHE and starts increasing exponentially above 2.0 V versus RHE.

HERFD–XANES measurements were performed at the ID26 beamline (Gauthier *et al.*, 1999 ▸) of the European Synchrotron Radiation Facility, in Grenoble, France. A double-crystal Si(311) monochromator was used. Higher-order harmonics were rejected using three Cr/Pd mirrors at an angle of 2.5 mrad relative to the incident beam. XANES spectra were measured simultaneously in total fluorescence yield (TFY) mode using a photodiode and in HERFD mode using an X-ray emission spectrometer in the Johann geometry (Glatzel *et al.*, 2021 ▸). The Bi *L*_III_-edge HERFD–XANES spectra were obtained by recording the maximum intensity of the Bi *L*_α1_ emission line as a function of the incident energy while the V *K*-edge ones were recorded by measuring the intensity of the V *K*_α_ line. In both cases, the intensity was normalized to the incident flux.

**Figure 2:**
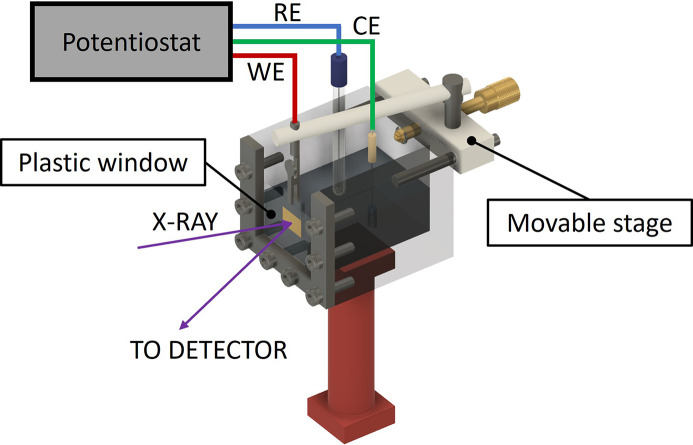
Sketch of the custom-made electrochemical cell for operando measurements. RE = reference electrode, WE = working electrode, CE = counter electrode.

In order to perform operando HERFD–XANES measurements we designed a custom electrochemical cell, illustrated in Fig. 2 ▸. The body of the cell is made of PMMA (polymethyl methacrylate) and consists of a reservoir filled with the electrolyte (0.5 *M* Na_2_SO_4_, neutral pH), in which the reference [saturated calomel electrode (SCE)], counter (Pt) and working electrodes are accommodated. The conversion between potentials is: *E* (V) versus RHE = *E* (V) versus SCE + 0.24 + (0.059 × pH). In particular, the working electrode is held in position by an alligator clip mounted on a movable stage, allowing the sample to be translated as close as possible to the transparent window to minimize X-ray attenuation due to the absorption of the electrolyte layer between the sample and the window. The transparent window is made of a 150 µm-thick acetate foil. The cell was controlled by an Autolab PGSTAT204 potentiostat.

## 3. Results and discussion

We first present and discuss the HERFD–XANES spectra acquired at the V *K*- and Bi *L*_III_-edges in open circuit potential (OCP) conditions and subsequently the changes observed upon application of a potential. We note that we have previously published the OCP spectra along with a comparison with literature XANES spectra of BiVO_4_ and *ab initio* spectral simulations using *FDMNES* (Cristino *et al.*, 2019 ▸). Here we briefly discuss these spectra in comparison with those of reference oxides (Mistonov *et al.*, 2018 ▸) and electronic structure calculations of BiVO_4_ (Cooper *et al.*, 2014 ▸).

**Figure 3:**
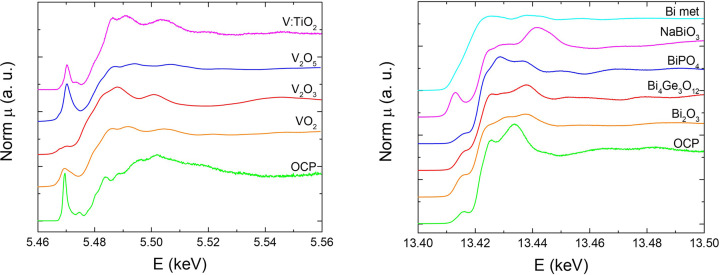
HERFD–XANES spectra at the V *K*- and Bi *L*_III_-edges (left and right, respectively) in the BiVO_4_ photoanodes in OCP conditions and in selected reference compounds, as described in the text.

The spectra acquired in OCP conditions are reported in Fig. 3 ▸, along with those of selected reference compounds. The V edge spectra are compared with HERFD–XANES spectra of substitutional V in rutile nanoparticles, labelled as V:TiO_2_ (Rossi *et al.*, 2017 ▸), and with (non-HERFD) XANES spectra of V_2_O_3_, VO_2_ and V_2_O_5_ (Rossi *et al.*, 2016 ▸). The overall lineshape in the BiVO_4_ photoanode does not resemble closely any of the other spectra. However, we note that the intensity of the pre-edge peak is highest in BiVO_4_ and is close to that of V_2_O_5_. The intensity of this peak is known to scale with the oxidation state of V (Bordage, 2010 ▸), so this finding is compatible with the expected 5+ oxidation state of V in BiVO_4_. The Bi *L*_III_-edge spectra for BiVO_4_ are similar, but not identical, to the spectrum of Bi_4_(GeO_4_)_3_, again compatible with the expected 3+ oxidation state.

**Figure 4:**
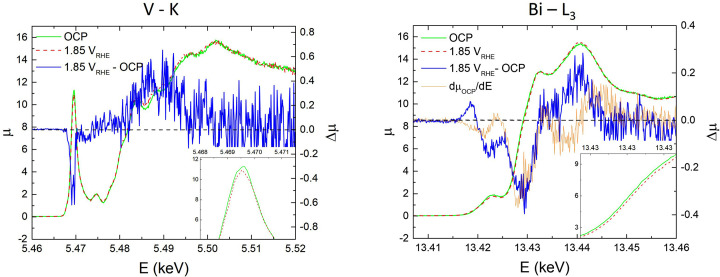
HERFD–XANES spectra at the V *K*- and Bi *L*_III_-edges (left and right panels, respectively) in open circuit conditions (green) and upon application of 1.85 V versus RHE potential (dashed red); the insets highlight the changes for the pre-edge of the V edge spectrum and for the rising edge of the Bi *L*_III_-edge one. For both edges the difference curves (+1.85 V versus RHE applied potential minus OCP) are reported in blue and the corresponding ordinate is on the right. For the Bi *L*_III_-edge spectrum the derivative of the OCP spectrum is also reported in orange.

In Fig. 4 ▸ we compare the HERFD–XANES spectra at both the V *K*- and Bi *L*_III_-edges in OCP conditions and upon the application of a +1.85 V versus RHE potential. Difference curves (application of the potential minus OCP) and, for the Bi *L*_III_-edge data, the derivative of the OCP spectrum are also reported. The small differences observable in the raw data are clearly well above the noise level, as shown by the difference curves. Specifically, the intensity of the V pre-edge peak (1*s* to 3*d* transitions to the bottom of the conduction band) clearly decreases and there is a small increase of parts of the main edge. A reasonable explanation of this observation is that it is due to the migration of negatively charged SO_4_^2−^ anions from the solution and their adsorption on the BiVO_4_ surface induced by the positive potential. The excess charge carried by the anions partially fills the 3*d* states at the bottom of the conduction band, which are highly localized on V (Cooper *et al.*, 2014 ▸), leading to a reduction of the pre-edge peak; we hypothesize that these charges remain trapped in the near-surface region and do not flow in the circuit. We note that this effect on the V edge spectra is different from that caused by illumination of V-doped TiO_2_ previously reported by our group (Rossi *et al.*, 2017 ▸); in that case we observed a rigid shift of all the spectrum, which we attributed to a light-induced electron transfer from V to Ti.

The Bi *L*_III_-edge, instead, is rigidly shifted to higher energies, in analogy to an oxidation. Even if the applied potential is not sufficient to intercept the valence band of the semiconductor, the maximum of which is located at about +2.5 V versus RHE (Sivula & van de Krol, 2016 ▸), the enhanced band bending Δϕ increases the density of surface holes according to *p*_s_ 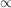 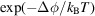 . Therefore, we can hypothesize that the removal of electrons from states at the top of valence band associated with O 2*p* and Bi 6*s* results in a net decrease of the charge on Bi cations giving rise to the observed positive shift of the Bi *L*_III_-edge.

In conclusion, the data reported highlight the ability of operando HERFD–XANES in an electrochemical cell to simultaneously detect small but significant changes of the spectra at both cation absorption edges in BiVO_4_ photoanodes. We interpret the observed variations induced by changes of the applied potential in terms of changes of the occupation of electronic states at or near the band edges localized on the two cations.
